# Erysipelas Treated by Severe Cold or Congelation: With Remarks on the Superiority of This Agent as a Remedy for External Inflammation

**Published:** 1849-09

**Authors:** 


*Erysipelas Treated by Severe Cold or Congelation: with Remarks on the Superiority of this Agent as a Remedy for External Inflammation.* By JAMES ARNOTT, M. D.

The congelation or freezing of the animal textures produced by powerful frigorific mixtures, may be considered in its threefold character of a remedy, a prophylactic, and anæsthetic, or preventive of pain in surgical operations.

Congelation is a remedy of many diseases affecting the nervous and vascular systems. Of external inflammation it is a certain, speedy, safe, and agreeable remedy.

Certain, because wherever congelation can be produced, inflammation ceases. Every other remedy of inflammation, as blood-letting, antimony, mercury, minor degrees of cold, &c., are more doubtful in their effects.

Speedy, because congelation instantly arrests inflammation. The congestive state which sometimes succeeds, has nothing of the character of inflammation, and none of its consequence. Where the degree of duration of the refrigeration has been insufficient, or where the cause of disease continues to operate, the inflammation will, after a considerable period, return; but a re-application of the remedy will again immediately arrest it.

Safe, because in no instance, of hundreds in which it has been employed, has congelation been productive of any injury or untoward effect. Blood-letting often proves destructive, by prostrating the vital power required for reparation; and the other remedies have all their respective evils or dangers. Still, as every other potent remedy may be abused, so might congelation prove prejudicial if too long continued, or if produced by frigorific mixtures of greater power than is required. In some cases it may be proper that congelation should be followed by the application of the "current apparatus," or the means which I have introduced for regulating local temperature with precision, in order to obviate reaction of the deeper tissues.

Agreeable, because it is speedy,—because it instantly benumbs the part, and relieves the pain accompanying inflammation. Excepting a slight tingling when the congelation commences, and for a few minutes after its cessation, this therapeutical agent causes no unpleasant sensation; such as the pain from the operations by which blood is extracted, or the fainting thus produced; the nausea and vomiting from antimony; the soreness of the mouth from mercury; the pain from scarification in phlegmonous erysipelas, &c., &c.

The prophylactic virtue of congelation is the power which it possesses of preventing inflammation of parts which have been subjected to its influence. Wounds produced by surgical operations (as already stated in my paper in the *Medical Gazette* of December 1st) have invariably appeared to heal more speedily after the application of congelation, than under the usual circumstances, and probably on account of the absence of any injurious degree of inflammation. Indeed, it was the observation of this effect of congelation in preventing inflammation, which led to its use as a remedy of the same condition; and conversely, had it been first used as a remedy, its preventive power would probably have been as soon discovered. This property of preventing injurious vascular excitement ought alone, and independently of its anæsthetic virtues, to render the use of congelation a preliminary to surgical operations, for even the smallest of these occasionally proves fatal, in consequence of inflammation. A sad illustration of this has recently been afforded by the lamented death of a very distinguished statesman, who fell a victim to the consequences of a very trifling operation performed to remedy an inconvenience so slight that it could scarcely be called disease.

The third medical property of congelation, is its power of preventing pain in surgical operations. Its excellence in this respect, compared with ether or chloroform, consists first, in its power of producing local anæsthesia while the consciousness of the patient remains undisturbed; and secondly, and especially, in its perfect safety. Since the publication of my former remarks on this subject, other *sudden* deaths from chloroform have been reported by the press: of *eventual* fatal consequences and other mischies there is no record.

The remedial powers of congelation in inflammation are proved by the following cases of erysipelas, in which it was employed:—

To the philosophic physician, acquainted with the history of the treatmen of erysipelas, the announcement of a new remedy for it will probably at first appear only as another example of the common fallacy of attributing the cure of a disease to the use of a medicine or remedial means, merely because the disease ceases after it has been administered. But there is this essential difference between the means now recommended, and the numerous and diversified expedients hitherto resorted to in erysipelas, that the former has in almost every instance in which it had been employed produced an immediate and very obvious beneficial effect; whereas the latter, it will be generally admitted, have just as frequently appeared to be inert or injurious, as to be efficient and useful.

Congelation, in respect to its use in erysipelas, is what is termed a rational remedy. Its analogies with other acknowledged remedies of inflammation would recommend its employment in this disease. Much of the danger of the erysipelas which affects the face and neck, unquestionably proceeds from the extensive and severe inflammation of the skin; and to the suppression of this the efforts of physicians have been directed. Now, as cold is a remedy of inflammation of admitted efficacy, it is reasonable to suppose that by subjecting the diseased tissue, and this alone, to a short application of a much greater degree of cold than has hitherto been employed, a greater depressing or antiphlogistic power may be exerted. Again, as experience would show that bleeding when it produces syncope is a more certain mode of checking inflammation than when it does not produce that effect, so severe cold or congelation, which, like fainting, checks the circulation of blood through the part subjected to it, may likewise be useful, for the same reason, and under the same circumstances. The morbid action of the blood vessels being thus arrested for a time, the healthy circulation may, by the efforts of nature, be immediately afterwards restored. Such reasonings, however, are of little importance in comparison with the following facts:—

Case I.—Charlotte Shepherd, 10 years of age, living at 17, New Dorset street, became a patient of the Brighton Dispensary on the 15th of Nov., 1848. When I first saw her, two days afterwards, there was much swelling and redness of the face, and the eyes were closed. Considerable fever was present, and, occasionally, delirium. She had been purged, and had taken antimonial and saline medicines without any mitigation of the symptoms. I applied a mass of pounded ice and salt, by means of a flat sponge, to each side of the face, for about a minute, or until large patches of the skin had become white and hard, or in other words, frozen. She did not complain of the application, but on the contrary-appeared to obtain immediate relief. The salt was washed off the face, and the saline mixture ordered to be continued.

17th.—The erysipelas has extended to the neck, and has returned to one side of the face and the ear. Increase of delirium and of the general febrile symptoms. The frigorific was again applied as before, to the inflamed surface, and with the same immediate beneficial result. To take a laxative, and to continue the mixture.

18th.—The fever and delirium subsided towards the evening of yesterday. The swelling has now quite left the face, and nearly the neck.

From this period the convalescence was rapid. Little medical treatment, besides attention to diet, was deemed necessary during the remaining period of attendance.

A younger sister of this girl was attacked with erysipelas about a month afterwards, and died after a fortnight's illness. The fever was typhoid, and she gradually sunk from exhaustion. She was judiciously treated by moderate antiphlogistic remedies in the first, and by tonics and stimulants in the latter stage. My opinion was requested towards the end; but I did not think that congelation could then be of service. I now regret that it was not employed, as, without putting the patient to the least hazard, it would have removed or lessened one cause of asthenia, and diminished one source of suffering.

Case II.—W. Mansfield, aged 47, residing at No. 1, Leicester street, admitted a patient of the Dispensary, with erysipelas, on the 12th January, 1849. Was seen at first by the house-surgeon, who prescribed a laxative, and a saline mixture containing antimony. When I took charge of the case on the 14th, I found him laboring under the disease in its severest form. He had been very delirious during the night, and continued to be excited, and at times incoherent. The face was much swelled and distorted, and the eyes closed. He complained of a very painful sense of burning in the inflamed parts. There was much fever. I applied pounded ice and salt in a piece of thin silk gauze, to the whole of the inflamed surface, by shifting the bag from place to place to place, and with the effect of freezing large patches of the skin. Each application may have lasted nearly two minutes. There was a little smarting during and immediately after the congelation, but this was succeeded by complete relief. To continue the medicine already prescribed.

15th.—The inflammation on the face hardly perceptible, but it has extended all round the neck and the pain is severe. Passed a restless night, and the fever, which had subsided for about twelve hours, again rose to its former height. The frigorific was again repeated, and kept in contact with the different portions of the inflamed skin, until nearly the whole had become white and frozen. A mixture containing quinine to be substituted for the saline medicine.

16th.—Little appearance of inflammation on any part of the face or neck, and no uneasiness. Slept better in the night, though occasionally incoherent.

The fever has decreased. To continue the tonic, and to take wine.

From this time, and under the same tonic remedies, he recovered rapidly.

Case III.—Harriet Tree, aged 4 years, residing at No. 16 New Dorset street, (next door to the residence of the girl whose case has been related) was admitted a patient of the Dispensary, with fever, on the 15th January, under the care of Mr. Smith, who obligingly transferred the case to me when the inflammation of the face had betrayed the nature of the disease.

On the 16th, inflammation was perceptible on both the cheeks and the forehead. I applied to these parts in succession a solid bit of ice, covered with salt, and which had been slightly hollowed into corresponding shape, by being held for a few seconds in contact with a jug containing hot water. The refrigoration thus produced was not sufficient to blanch the skin, tho' the application was made for more than a minute. A saline mixture containg autimony and hyoscyamus, to be continued.

17th.—The inflammation has spread over the face, and the eyes are opened with difficulty. Pulse febrile; restless, and apparently in much pain. A mixture of ice and salt was applied over the inflamed surface, but with little more apparent effect than on the former occasion, owing probably more to the insufficient quantity of the frigorific employed, than to the violence of the inflammation. A mercurial laxative to be taken night and morning, and the fever mixture continued.

18th.—Much ease appeared to have been afforded by the applications of yesterday, as she slept soundly for some time afterwards, and had immediately ceased complaining. The uneasiness, however, returned in the night, and she is now ( 3 P. M.) very restless, and raising her hand incessantly to her face, on which there are several vesications. The frigorific re-applied for about a minute, and had the effect of freezing portions of the skin After the skin had been washed, she was placed in bed and almost immediately fell asleep.

19th.—Better. The face is still swelled, and the eyes shut; but there is less heat in the inflamed part, and less fever. To continue the medicines.

20th.—The neck now much inflamed, as well as both sides of the face and both ears. The fever has increased. Tongue dry, with a brown fur in the centre: and the mouth appears inflamed. In the evening the frigorific was again applied, and much more effectually than on former occasions. About a pound of ice having been well pounded, (in a small canvas bag, placed on the brick floor, by means of a flat-iron,) and quickly mixed with about half the quantity of salt, was put into a thin silk gauze bag, and applied for upwards of a minute over the face and neck, a third or fourth portion of the surface at the time; the frigorific being renewed for the last applications, and the melting ice being absorbed by by cloths placed close to the bag or net containing it. The whole surface was thus frozen, and continued hard and white for half a minute. A mixture of acetate of ammonia and soda prescribed, and taken at intervals.

21st.—Much relief was given by the congelation, as on the former occasion, and the swelling of the face and neck has nearly disappeared, excepting the eyelids, which continue closed. Tongue dry and more furred. Pulse more frequent, and much restlessness. The sensorium continues unaffected. Refuses to take food, and has evidently pain from what is forced upon her, and a difficulty of swallowing it. The breathing is not embarrassed, and there is a frequent hacking cough. To have wine and beef tea.

22d.—The inflammation has not returned to the neck or face, but appears to have increased in the mouth and fauces. Tongue very foul and dry. Pulse quick and weak. Occasional incoherence. To continue the wine, and to take a mixture containing carbonate of ammonia every four hours. An opiate at bed-time.

23d.—Better. Tongue more moist; less restlessness; takes nourishment more willingly. A laxative prescribed.

24th.—Recovering. When she was at last able to open her eyes, the conjunctiva of one was observed to be much congested; but there was no intolerance of light, or expression of uneasiness from this cause.

It will have been remarked in perusing the details of the above cases, that the beneficial effects of congelation were immediate, and otherwise so well marked as to prevent any doubt of its efficiency. In this respect it is strikingly in contrast with the remedies hitherto employed in erysipelas. The practice of scarifying the inflamed surface, or puncturing all over with a lancet may frequently be of some service, notwithstanding the irritation which the wounds themselves, and their exposure to the air, must necessarily produce; but the painting of the part with lunar caustic, and the application of warm fomentations, or cold lotions, I am disposed from my own observation, to place, with respect to efficiency, in the same category with the old practice of the application of flour.

The absence of all injurious effect, or untoward consequence, from the congelations that were used, will also be equally obvious. The cerebral disturbance was uniformly relieved, and had the patient whose case is last related been of more advanced age, so that a solution of salt about the temperature of zero might have been easily applied to the mouth and fauces, her disease, I have little doubt, would have had an earlier termination. A stronger application of congelation to the face might, perhaps, have had a similar effect, by preventing the extension of the inflammation to the mucous surface.

The applications of severe cold were generally slight, and I am inclined to think that they would have been more efficacious had they been less so; that there might, at least, have been less necessity for repeating them. But there is, probably, no great difference, as respects the safety of the patient, between at once removing the inflammation and the susceptibility of its renewal, and checking it again and again on its approach; unless, indeed, the disposition to spread, just adverted to, be thus prevented. Some of the applications were milder than was desirable, on account of a defect in the means employed. If ice and salt be the frigorific resorted to, it is proper, where the skin is acutely inflamed, and consequently greater frigorific power is required, to employ it in the best and most effectual manner, as on the last occasion of its being applied in the case. The greater expenditure of material now that ice can be every where procured at trifling cost, and in every season, is a point of no importance.

Although congelation may have no power in shortening the period of erysipelatous fever, or preventing it running through its several stages, (and it certainly did not appear to have this power in the third case related,) it will obviate the danger that would arise from the accompanying external or accessible inflammation. The danger from small-pox is, *cœteris paribus*, very much in proportion to the extent and degree of the inflammation of the skin, and particularly, in the opinion of Sydenham, of the skin of the face. It is this, probably, which makes the great distinction, in respect to danger, between the distinct and confluent species; and the same principle probably applies to erysipelas. A high and extensive inflammation must (as has likewise been remarked by Sydenham) necessarily increase the febrile action in this disease, or cause, as it were, a symptomatic fever in addition to that, which is specific, and tend to exhaust the animal powers:—tend, in fact, to produce or aggravate the asthenia which in erysipelas is usually the cause of death. Inflammation of other systems or organs, occurring in typhus and other febrile diseases, must for the same reason, and independently of any consequent disorganization, materially increase their danger; but in all such cases, whether the skin or internal organs be affected, there is, in addition, the irritation or injurious influence proceeding from the disturbance of the function of the inflamed part. It is, therefore, not only in erysipelatous fevers that congelation will be found a remedy of great importance in subduing local affections; it will probably be also very serviceable in other analogous diseases accompanied with inflammation of superficial or other accessible parts. The skin, mouth, and throat, are obviously under its control; the windpipe and cerebral membranes are not probably beyond its reach. If the latter do not admit of congelation, they may have their temperature reduced to a much greater degree than has been hitherto attempted, and with great remedial advantage. The point to be aimed at is, perhaps, not so much congelation, as that degree of refrigoration which will *permanently* depress the nervous and vascular energies, or depress them without causing reaction. This must be far below the degree to which any application of water or ice will reduce the temperature of the part.

The notion that certain external inflammations are, even to their full existing amount, necessary safety valves or emunctories for the materies morbi, or are otherwise essential to the patient's safety, is now, happily, nearly exploded. Physicians have become well aware that hypothesis, or ill-founded theory, has formed the grand impediment to the progress of the art of healing; and in no instance has the superiority of observation to theory been more remarkable than in the modern treatment of erysipelas. Amongst other means of subduing the external inflammation, cold applications are now generally recommended; and in this improvement there is only a return to the practice of Celsus (Book v. cap. 26) and of his successors for many ages. In confirmation of the downfall of the doctrine of metastasis from cold, the public lectures on the practice of physic of two distinguished professors in the colleges of the London University, and the lectures of the present occupants of the chairs of surgery in the medical school of St. Bartholomew's Hospital and University College, may be referred to. "There is no hazard," says Dr. Watson, speaking of the use of cold in erysipelas, "such as you may read of, of the inflammation being repelled from the surface and driven in upon some vital organ." But even granting that cold, as it has hitherto been usually applied, is dangerous in certain specific inflammations from its tendency to cause the metastasis, (and there is little authority in favor of its use in rheumatism or gout,) it must be especially borne in mind, that there is a wide difference between congelation and such applications of cold. At first it might appear to differ only in degree,—in being greater than these and consequently more dangerous; but in truth, it is much less a cooling application. If a physician wishes to heat a limb of a patient, he would surely have it immersed for half an hour in warm water, in preference to applying a red hot iron for a few seconds to the skin; and he would in cooling a limb make a similar distinction between continued cold and momentary freezing. Dangerous as plunging a limb affected gout into cold water, according to Harvey's plan, may be, the same objection would not apply to the exactly limited and short application of congelation to the affected part; and the same observation would apply to the treatment of many varieties of rheumatism. Analogy, on the contrary, would point out such a remedy as one likely to be eminently useful in these complaints, in allaying suffering and preventing the disorganization of joints, while appropriate medicines were simultaneously exhibited as antidotes to, or evacuants of, the supposed materies morbi.

The resemblance between erysipelas and the exanthemata, or eruptive fevers, may have led to the unfounded fear of metastasis from cold; but although there are resemblances between these diseases, there are great differences as well: the frequency with which the same individual may be attacked by erysipelas is one of these; and the irregularity of the course of the disease, (supposing that there is only one kind of erysipelatous fever—a point by no means established,) is another. I believe that much more importance is attributed to the cutaneous eruption in diseases of this class than ought to be. If there be poison eliminated, there are other emunctories for this purpose besides the skin. As there is frequently scarlatina without eruption, so probably, measles and the fevers of erysipelas may exist without it. Nay, considering that cases of small pox occasionally occur with scarcely a dozen pustules spread over the skin, it is not very improbable that even this disease, (as was, indeed, the opinion of Sydenham,) may likewise run its course, and the system be purged of its poison, without observable cutaneous affection. In the fever caused by vaccination there is no eruption of pustules; yet, if there be poison evacuated in the natural small-pox, it can scarcely be doubted that an analogous process takes place in cow-pox, which is only a modification of it. The immunity which many persons appear to possess from the contagions of the several exanthemata would thus readily admit of explanation; and more effort might be made (were this point established) to endeavor that the disease should run a milder or safer course, instead of "forcing the eruption out," or causing extensive and assuredly dangerous inflammation of the skin, and suspension or derangement of its important functions.

The necessity of repeating the congelations in the cases narrated above, may have been partly owing to the insufficient degree of many of the applications, and partly to the still existing cause of the inflammation—the exanthematous fever. While the cause of the inflammation continues active, a great change indeed would be required in the organization or function of the part to prevent its return. I do not say, that this cannot be effected by congelation, but when the application can be so easily renewed, without pain or discomfort to the patient, such a change does not apear very important. When congelation has been used to arrest the inflammation and suffering which arise from mercurial ptyalism, (and no remedy of this di tressing state is comparable to congelation in efficacy and celerity of action,) it has been usually necessary to repeat the application after an interval, on account of the persistence of the cause; and as the application of the frigorific immediately benumbs, the patient has never objected to its repetition. In suppurating boils, carbuncles and glandular swellings, when congelation has been used to put a stop to the suffering of the patient and procure sleep, it has been seldom necessary to repeat the application, notwithstanding the persistence of the cause, probably on account of the inflammation being thereby much subdued; and the formation of pus being rendered much slower, the parts are more adapted to it—the fibres have become not only less sensitive, but they are less stretched. In some instances the matter appeared to have become more absorbed, as the swelling gradually disappeared without breach of substance. It would generally, however, be a better practice in these cases, seeing that after a momentary congelation the abscess can be opened without pain, at once to liberate the purulent deposit and relieve the distant fibres by a free incision.

I trust that enough has been said to induce practitioners to have recourse to congelation in the treatment of erysipelas. Conscious that a strong prejudice exists against the employment of extreme cold, from the error of not discriminating between its effects when the body has been exposed for an unlimited period, and when it is employed remedially, and carefully limited in degree, duration and extent, I had almost determined not to write again on the subject until I had an opportunity of doing so fully, and with a host of experimental proofs of many allegations; but the conviction that congelation is the only remedy to be relied upon in erysipelas, and that by its use much suffering and many lives may be saved, has persuaded me to the publication of this paper. I have only had the opportunity of employing congelation in the three cases of erysipelas which I have related, but the results of its application in these are sufficient to establish the excellence of the treatment. And the publication of these proofs, not only of the perfect safety, but of the great advantage of congelation in a species of inflammation which is usually considered as prone to gangrene, cannot fail to remove the apprehension of injury from employing it under different circumstances, and for other and not less important purposes. With respect to its remedial properties, I shall, on another occasion, give illustrations of the use of congelation, in phlegmonous inflammations, chronic diseases of the skin, ophthalmia, &c.; and in certain neuralgic affections, including varieties of headache. Although headache may not often shorten life, it very often embitters it, by resisting every remedy that has been hitherto employed. In no disease has the efficiency, safety and speedy operation of congelation been more conspicuous than in this painful affection; which appears in many cases, to arise from a more or less permanently morbid state of the nerves of some portion of the forehead or scalp.—*Monthly* (*England*) *Magazine.*

